# Antiviral Agents, 2024

**DOI:** 10.3390/ph19101621

**Published:** 2026-10-13

**Authors:** Luis Octavio Regasini

**Affiliations:** Department of Chemistry, Institute of Biosciences, Humanities and Exact Sciences, São Paulo State University, Rua Cristóvão Colombo 2265, São José do Rio Preto 15054-000, Brazil; luis.regasini@unesp.br

Viruses are small infectious agents (20–400 nm) that are not able to replicate without host cells. They contain only one nucleic acid type (RNA or DNA) as their genome, surrounded by a protein envelope that may be delimited by a lipid membrane. There are approximately 25 viral families associated with human infections, highlighting Picornaviridae, Herpeviridae, Reoviridae, Togaviridae, Flaviviridae, Coronaviridae, Retroviridae, Orthomyxoviridae, Bunyaviridae, and Paramyxoviridae [1]. Of particular significance are (re)emergent viruses, including the Marburg, Bundibugyo, Hantavirus, Nipah and Yellow Fever, Measles, and Mpox viruses, which have been related to outbreaks in the current decade [2]. Considering that viruses use many host metabolic processes, it is difficult to find antiviral agents that are selective for the pathogen biomolecules. However, there are certain viral cycle points that are specific to viruses, and these have become valuable molecular targets in medicinal chemistry [3].

Antiviral agents include diverse compound classes with varied actions, which can act on all parts of viral cycles. From the first antiviral drug approved by the Food and Drug Administration, idoxuridine (a nucleoside analog) to nirmatrelvir (an anti-SARS-CoV-2 drug), the discovery of antivirals has experienced constant evolution [4,5]. In this context, phenotypic [6,7] and target-based approaches [8,9] have been central strategies for the search for innovative antivirals, which can be driven by contemporary artificial intelligence [10,11].

This Special Issue of Pharmaceuticals was devoted to state-of-the-art research on the discovery and development of natural and synthetic antiviral agents using in silico and in vitro experiments. The Special Issue contains five elegant research articles with complete descriptions of each study and the main results were presented in full respective manuscripts, which the readers are invited to read. A summary of the articles is presented herein.

In the first research article in this Special Issue, Albiheyri et al. used in silico tools to investigate specialized metabolites from *Nyctanthes arbo-tristis* Linn. (popularly named “night-flowering jasmine”, “parijat” and “tree of sadness”) against SARS-CoV-2 (Coronaviridae), Ebola (Filoviridae), Nipah (Paramyxoviridae), and Chikungunya (Togaviridae) proteins (Contribution 1). Virtual screening and AutoDock 4.2 calculations demonstrated that arborside C (an iridoid glycoside), β-sitosterol (a sterol), β-amirin (a pentacyclic triterpenoid), and nicotiflorin (a flavonoid glycoside) exhibited substantial binding affinity for viral proteins. Arborside C and β-sitosterol had binding energies of −8.65 and −9.11 kcal mol^−1^, respectively, when compared to remdesivir (an anti-COVID-19 drug; −6.18 kcal mol^−1^) in the main protease of SARS-CoV-2 (M^pro^, PDB ID: 6LU7). Β-Amirin displayed the highest binding energies in VP35 of Ebola (PDB ID: 4IBB) (−7.52 kcal mol^−1^) and nsP2 protease of Chikungunya (PDB ID: 3TRK) (−8.54 kcal mol^−1^). Finally, molecular dynamics simulation data corroborated a stable pattern for the β-amirin–VP35 complex, evidencing its anti-Ebola potential.

The article by Lai et al. presented the in vitro antiviral effects of *Rhodiola crenulate* extract (RCE) and its specialized metabolites, including salidroside (phenylethanoid glycoside), tyrosol (a phenylethyl alcohol), and gallic acid (a phenolic acid) (Contribution 2). RCE and isolated compounds were assayed against two Flaviviridae viruses, which are causative agents of Zika (ZIKV) and Japanese Encephalitis (JEV). RCE exhibited anti-ZIKV and dose-dependent-JEV effects at non-cytotoxic concentrations, inhibiting ZIKV and JEV growth in the early infection stage, as well as suppressing ZIKV and JEV infection by viral binding inhibitory action. Among the tested compounds, gallic acid was able to explain the antiviral activity of RCE. Using the qRT-PCR technique, gallic acid was also found to reduce ZIKV and JEV RNA levels in Vero cells in dose-dependent manner.

In the study by Ding et al., the authors designed and synthesized novel p97 inhibitors (also known as valosin-containing protein, VCP) as promising antiviral agents (Contribution 3). In general, p97 has been recognized as a promising anticancer target. Additionally, it has a central role as pro-viral host factor and its inhibitors can be valuable antiviral agents. Among indole compounds, two displayed potent anti-p97 activity, including piperidine (**9a**) and cyclohexyl (**10a**) derivatives, with >90% inhibition at 0.10 µmol L^−1^. The authors performed docking calculations to clarify the binding mode of p97 inhibitors, using the cryo-EM structure of hexameric human p97 (PDB ID 5FTK), which contains subunits A–F. In silico experiments evidenced a covalent bond established between the bioactive compounds and Cys522 of the active site of the D2 domain. In in vitro assays, compounds **9a** and **10a** showed anti-SARS-CoV-2 activity with EC_50_ values of 6.7 µmol L^−1^ and 6.8 µmol L^−1^, respectively. Moreover, **9a** and **10a** had CC_50_ values of 76 µmol L^−1^ and >100 µmol L^−1^ on A549 (lung cells), respectively.

In their original research article, Ivanova et al. explored the broad-spectrum antiflaviviral activity of natural and synthetic compounds that were previously active against Dengue virus serotypes 2 and 4 (DENV-2 and DENV-4) (Contribution 4). Compounds **C1** (lycorine, a galanthanic alkaloid) and **C3** (mycophenolic acid) were active against all tested Flaviviridae viruses, including ZIKV, Kunjin virus (KUNV), and Tick-borne encephalitis virus (TBEV). Their EC_50_ ranges (in µmol L^−1^) were 0.13–0.53, 0.17–0.21, and 0.09–0.61 against ZIKV, KUNV, and TBEV, respectively. Altogether, molecular docking studies and replicon-based tests corroborated NS5 RdRp (RNA-dependent RNA-polymerase) and 2K-NS4B (cleavage protein) as molecular targets for **C3** and **C1**, respectively.

The final research article in this Special Issue by Li et al. reported the synergistic mechanisms between arbidol (an antiviral drug) and compounds from Toujie Quwen Granules (TQG, a polyherbal Chinese formulation) against M^pro^ SARS-CoV-2 (PDB ID 6LU7) (Contribution 5). Virtual screening and dynamic simulations were conducted on five promising compounds, which were able to synergistically act with arbidol in M^pro^. However, untargeted metabolomic investigations of TQG identified only baicalein (a flavonoid) as a specialized metabolite with ideal pharmacokinetic and non-toxic properties. Longer dynamic simulations revealed *“Far” Addition Mechanism #2* with a 2:1 stoichiometric ratio of arbidol to baicalein. The main reason for the synergistic mechanism was the reduction in arbidol movement by diffusion and intramolecular conformational shifts caused by baicalein.

In brief, this Special Issue collected relevant contributions on different classes of natural and synthetic compounds and their impact on viral protein targets by using in silico and in vitro approaches. The objectives of this Special Issue have been successfully achieved; for this reason, I would like to express my heartfelt appreciation to all authors, reviewers, and editors involved for their excellent contributions.

## Data Availability

No new data were created or analyzed in this study. Data sharing is not applicable to this article.

## Abbreviations

The following abbreviations are used in this manuscript:

DENV-2	Dengue virus serotype 2
DENV-4	Dengue virus serotype 4
JEV	Japanese encephalitis virus
KUNV	Kunjin virus
Mpox	Monkeypox virus
M^pro^	Main protease
NS5 RdRp	Non-structural protein 5 (NS5) RNA-dependent RNA-polymerase
PDB ID	Protein data bank identifier
qRT-PCR	Quantitative reverse transcription polymerase chain reaction
RCE	*Rhodiola renulate* extract
SARS-CoV-2	Severe Acute Respiratory Syndrome Coronavirus 2
TBEV	Tock-borne encephalitis virus
TQG	Toujie Quwen granules
VCP	Valosin-containing protein
ZIKV	Zika virus
